# Modulation of the CD95-Induced Apoptosis: The Role of CD95 N-Glycosylation

**DOI:** 10.1371/journal.pone.0019927

**Published:** 2011-05-18

**Authors:** Olga M. Shatnyeva, Andriy V. Kubarenko, Claudia E. M. Weber, Alexander Pappa, Reinhard Schwartz-Albiez, Alexander N. R. Weber, Peter H. Krammer, Inna N. Lavrik

**Affiliations:** 1 Division of Immunogenetics, German Cancer Research Center, Heidelberg, Germany; 2 Junior Research Group “Toll-like Receptors and Cancer”, German Cancer Research Center, Heidelberg, Germany; 3 Division of Translational Immunology, German Cancer Research Center, Heidelberg, Germany; National Institute for Medical Research, Medical Research Council, London, United Kingdom

## Abstract

Protein modifications of death receptor pathways play a central role in the regulation of apoptosis. It has been demonstrated that O-glycosylation of TRAIL-receptor (R) is essential for sensitivity and resistance towards TRAIL-mediated apoptosis. In this study we ask whether and how glycosylation of CD95 (Fas/APO-1), another death receptor, influences DISC formation and procaspase-8 activation at the CD95 DISC and thereby the onset of apoptosis. We concentrated on N-glycostructure since O-glycosylation of CD95 was not found. We applied different approaches to analyze the role of CD95 N-glycosylation on the signal transduction: *in silico* modeling of CD95 DISC, generation of CD95 glycosylation mutants (at N136 and N118), modulation of N-glycosylation by deoxymannojirimycin (DMM) and sialidase from *Vibrio cholerae (VCN)*. We demonstrate that N-deglycosylation of CD95 does not block DISC formation and results only in the reduction of the procaspase-8 activation at the DISC. These findings are important for the better understanding of CD95 apoptosis regulation and reveal differences between apoptotic signaling pathways of the TRAIL and CD95 systems.

## Introduction

Apoptotic cell death is common in multicellular organisms and can be triggered by a number of factors including UV- or γ-irradiation, chemotherapeutic drugs and signaling from death receptors [1]. CD95 (APO-1/Fas) is a member of the death receptor family, a subfamily of the TNF-R superfamily [2]. Crosslinking of CD95 with its natural ligand CD95L (CD178) [3] or with agonistic antibodies such as anti-APO-1 induces apoptosis in sensitive cells [4]. In addition, triggering of CD95 induces a number of non-apoptotic activities [1], [5], [6].

The death-inducing signaling complex (DISC) is formed within seconds after CD95 stimulation [7]. The DISC consists of oligomerized CD95, the adaptor molecule FADD, two isoforms of procaspase-8 (procaspase-8/a and procaspase-8/b), procaspase-10 and c-FLIP_L/S/R_ [1], [8], [9], [10]. The interactions between the molecules at the DISC are based on homotypic contacts. The death domain (DD) of the receptor interacts with the DD of FADD, while the death effector domain (DED) of FADD interacts with the N-terminal tandem DEDs of procaspases-8, -10 and c-FLIP_L/S/R_. Procaspase-8 upon binding to the DISC undergoes oligomerization that results in processing of the zymogen, for which a two-step mechanism has been described. The first cleavage step generates the two subunits p43/p41 and p12 [11]. In a second cleavage step, the active enzyme subunits p18, p10 and the prodomains p26/p24 are produced. As a result the active caspase-8 heterotetramer p10_2_–p18_2_ is released into the cytosol to propagate the apoptotic signal [12]. The initial events of DISC formation and caspase-8 activation have not been clarified yet. Pre-oligomerization of CD95 *via* the Pre-Ligand Assembly Domain (PLAD) has been suggested to play an important role in apoptosis initiation [13]. Recently, there have been several new reports on X-ray structure of CD95 and FADD [14], [15], [16]. Although the reported X-ray structures contradict each other/are in disagreement in terms of the CD95/FADD structure, they provide a basis for consideration of the initial events preceeding caspase-8 binding and activation at the DISC.

Two CD95 signaling pathways have been identified so far [17]. Type I cells are characterized by high levels of CD95 DISC formation and increased amounts of active caspase-8 which activates downstream effector caspases-3 and -7. Type II cells are characterized by lower levels of CD95 DISC formation and, thus, lower levels of active caspase-8. In this case, signaling requires an additional amplification loop that involves the cleavage of the Bcl-2-family protein Bid by caspase-8 to generate truncated (t)Bid and subsequent (t)Bid-mediated release of cytochrome C from mitochondria. The release of cytochrome C from mitochondria results in apoptosome formation followed by activation of procaspase-9, which in turn cleaves downstream effector caspases.

**Figure 1:**
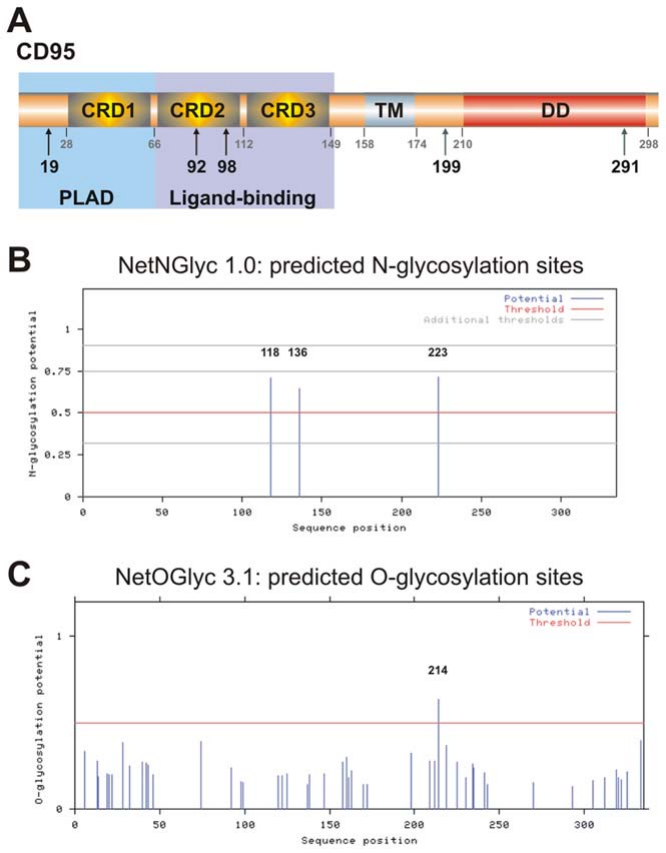
CD95 is a predicted glycoprotein. (A) Schematic representation of domain organization of CD95. The CRD are shown in yellow, the transmembrane domain (TM) in blue, the DD in red. Potential glycosylation sites are depicted as schematic oligosaccharides, phosphorylation sites as black arrows, palmitoylation sites as green arrows. (B) and (C) Prediction of N- and O-linked glycosylation sites in CD95. Graphical representation of prediction with indicated scores generated by server.

CD95 is a glycosylated type I transmembrane receptor (Figure 1A) and has been reported to be N-glycosylated in its extracellular domain [18], [19], [20]. N-linked glycosylation is introduced upon entry of the polypeptide into the lumen of the endoplasmic reticulum (ER) and involves the transfer of a carbohydrate moiety to an asparagine residue within a specific amino acid consensus sequence. In addition, CD95 was reported to be sialylated on the N-linked oligosaccharide chains [18], [19]. Sialic acids are a diverse family of sugar units with a nine-carbon backbone that are typically attached to the outermost ends of glycans [21], [22]. Sialylation is mainly regulated by sialidases and sialyltransferases, which cleave sialic acid residues from and transfer them to glycoconjugates, respectively [23]. It has been reported previously that desialylation of CD95 using *Vibrio cholerae neuraminidase* (VCN) results in increased sensitivity towards CD95-induced apoptosis [18], [19].

Glycosylation has been reported to play an important role in the modulation of the sensitivity towards death receptor-induced apoptosis. It has been reported that O-glycosylation of TRAIL-R is a major factor for the apoptosis induction. Further O-glycosylation promoted ligand-stimulated clustering of TRAIL-R1 and TRAIL-R2, which mediates recruitment and activation of procaspase-8 [24]. In this line, the aim of this study was to analyze the influence of CD95 glycosylation on apoptosis initiation and procaspase-8 activation at the DISC. Using amino acid sequence information and bioinformatic analysis we predicted that CD95 is N-glycosylated at N118 and N136. Furthermore, by means of *in silico* three-dimensional (3D) modeling we tentatively predict the possible mechanism of how N-glycosylation might influence DISC formation and procaspase-8 activation at the DISC. Surprisingly, on the experimental level, we could only find that deglycosylation of CD95 leads to the slowing down of procaspase-8 activation at the DISC. Notably, the formation of the DISC, *e.g.* the recruitment of FADD to the DISC was not blocked. The sensitisation upon CD95 N-deglycosylation took place only upon a narrow range of concentrations of CD95 antagonists. This demonstrated that, in contrast to the TRAIL-R O-linked glycan moiety, the CD95 N-glycan structure contributes to a smaller extent to the initiation of the apoptotic signaling leading to the death of the cells.

## Results

### Analysis of CD95 glycosylation using bioinformatic analysis and in silico 3D modeling

CD95 has been described to be an N-glycosylated protein [18]. To characterize CD95 N-glycosylation putative glycosylation sites of human CD95 were analyzed using bioinformatic analysis (Figure 1). The presence of several glycosylation sites was predicted, which is in accordance with previous reports and supports N-glycosylation of CD95 [20]. There are three N-glycosylation sites predicted (Figure 1B). Two N-X-S/T sites are located in the extracellular domains (residues 112–149) at positions N118 and N136 and one in the intracellular domain (174–298) at position N223 (Figure 1B). Predictions also show the presence of one O-glycosylation site at T214, which is highly unlikely as it is located in the CD95 intracellular domain (Figure 1C).

Analysis of an alignment of 16 sequences of CD95 from different species showed that the Asn residue in the first N-glycosylation site, which corresponds to N118 in human CD95, is the most conserved one. The Asn residue in the second N-glycosylation site, which corresponds to N136 in human CD95, is less conserved with regard to glycosylation (Figure S1A). Moreover, the N-X-S/T sequence of the second N-glycosylation site N136 is conserved in three organisms from all 16 analyzed, suggesting N136 residue could be potentially glycosylated (Figure S1B).

**Figure 2:**
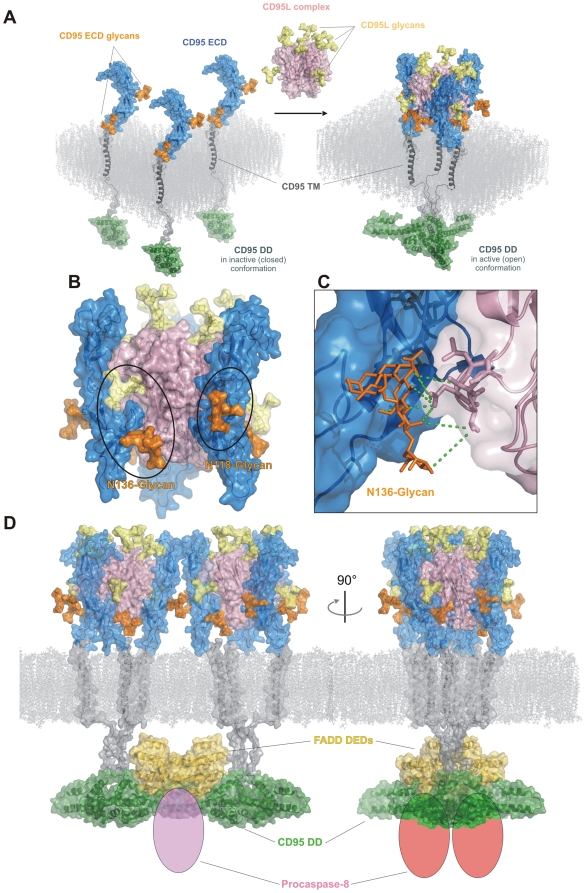
In silico modeling of the CD95 DISC and interactions of CD95 glycosylated residues. (A) Formation of CD95-CD95L trimer upon binding of CD95 ECDs to the CD95L ECDs trimer. (B) Close up on the interface between CD95 and CD95L ECDs. The glycan attached to N136 is in close proximity to CD95L. The glycan attached to N118 of CD95 is distal from the CD95L ECD. (C) The glycan attached to N136 of CD95 is in a close proximity to CD95L ECD and could form extensive hydrogen bonds (green dotted lines) with residues 200–204 of CD95L.

To analyze the possible role of CD95 glycans in the CD95 DISC formation and in the formation of the CD95 DISC complex network on the membrane *in silico* modeling was applied. Core structures of N-glycans were added using the GlyProt tool as presented in Figure S2A. It is generally accepted that CD95 DISC core structures are composed of three molecules of CD95 and three molecules of CD95L [16]. As depicted in Figure 2, upon formation of CD95 DISC core structure, the glycan attached to N136 of CD95 could potentially be important for complex formation and/or stability, due to its close proximity to CD95L molecule (Figure 2A, B) and could form an extensive hydrogen bond network with residues 200–204 of CD95L (Figure 2C). The glycan attached to N118 of CD95 most probably is not important for the formation and/or stability of the CD95 DISC core structure as it is located more distal from the CD95-CD95L interface (Figure 2B). On the other hand, the glycan attached to N118 of CD95 could be important for the stabilization of the DISC-DISC interaction upon formation of the CD95 DISC network. Oligomerisation of procaspase-8 might occur more efficiently and lead to more efficient procaspase-8 activation at the CD95 DISC network in the presence of CD95 glycans at N118. In this way, the modeling predicted a possible function of CD95 N-glycosylation for the proper caspase-8 activation (Figure 2D). We subsequently sought to validate the predictions of this modeling approach by further biochemical analysis.

### CD95 is N-glycosylated at two extracellular sites N118 and N136

**Figure 3:**
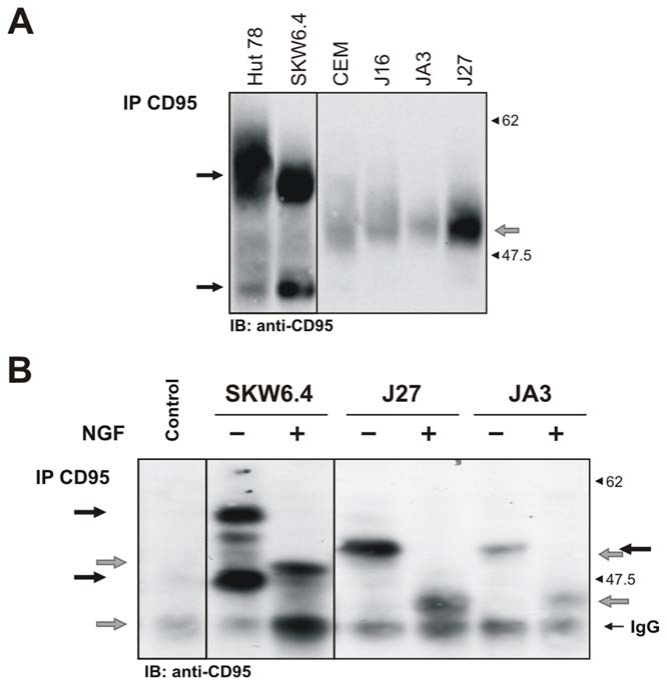
Analysis of CD95 glycosylation. (A) CD95 protein patterns were analyzed in the total cellular lysates of SKW6.4, Hut78, J27, JA3, CEM and J16 cells on 10% SDS-PAGE followed by Western Blot with anti-CD95 polyclonal antibodies C20. Two CD95 bands are indicated by black arrows, while one CD95 band is indicated by a grey arrow. (B) CD95 was immunoprecipitated from SKW6.4, J27 and JA3 cell lysates using anti-APO-1 antibodies. CD95-immunoprecipitates (CD95-IP) were subjected to N-glycosidase F (NGF) treatment with subsequent analysis by Western Blot with anti-CD95 polyclonal antibodies C20. Anti-APO-1 antibody alone was loaded on the same gel to control for the IgG bands (lane: control). Glycosylated bands of CD95 are indicated by black arrows, while deglycosylated bands are indicated by grey arrows. The band corresponding to the antibody chain is indicated. After enzymatic treatment with NGF the CD95 pattern was changed. In the case of the SKW6.4 cells the lower band of the CD95 pattern overlaps with the IgG_l_ upon deglycosylation.

To validate the bioinformatic predictions using biochemical analysis we sought to demonstrate that CD95 in our cell lines is indeed N-glycosylated. We compared CD95 in cellular lysates of different human T and B cell lines using anti-CD95 antibodies [25]. Interestingly, we observed that CD95 in B lymphoblastoid SKW6.4 and T leukaemia Hut78 cells appeared as two bands which are not the result of alternative splicing as assessed by RT-PCR (data not shown), while CD95 in T leukaemia cells J16 appeared as a single band on 10% SDS-PAGE (Figure 3A). The bands of CD95 had a diffuse and broad shape, which is characteristic for glycosylated proteins analyzed by Western Blot. To confirm N-glycosylation of CD95 we used N-glycosidase F, the enzyme, which cleaves off complete N-glycan moieties from a given protein (Figure S2B). The treatment with N-glycosidase F resulted in a clear shift of two major bands in case of SKW6.4 cells to a lower molecular mass range (Figure 3B). The one major band in case of J27 and JA3 cells also shifted to the lower molecular mass (Figure 3B). Therefore, we concluded that CD95 is N-glycosylated in all cell lines analyzed.

**Figure 4:**
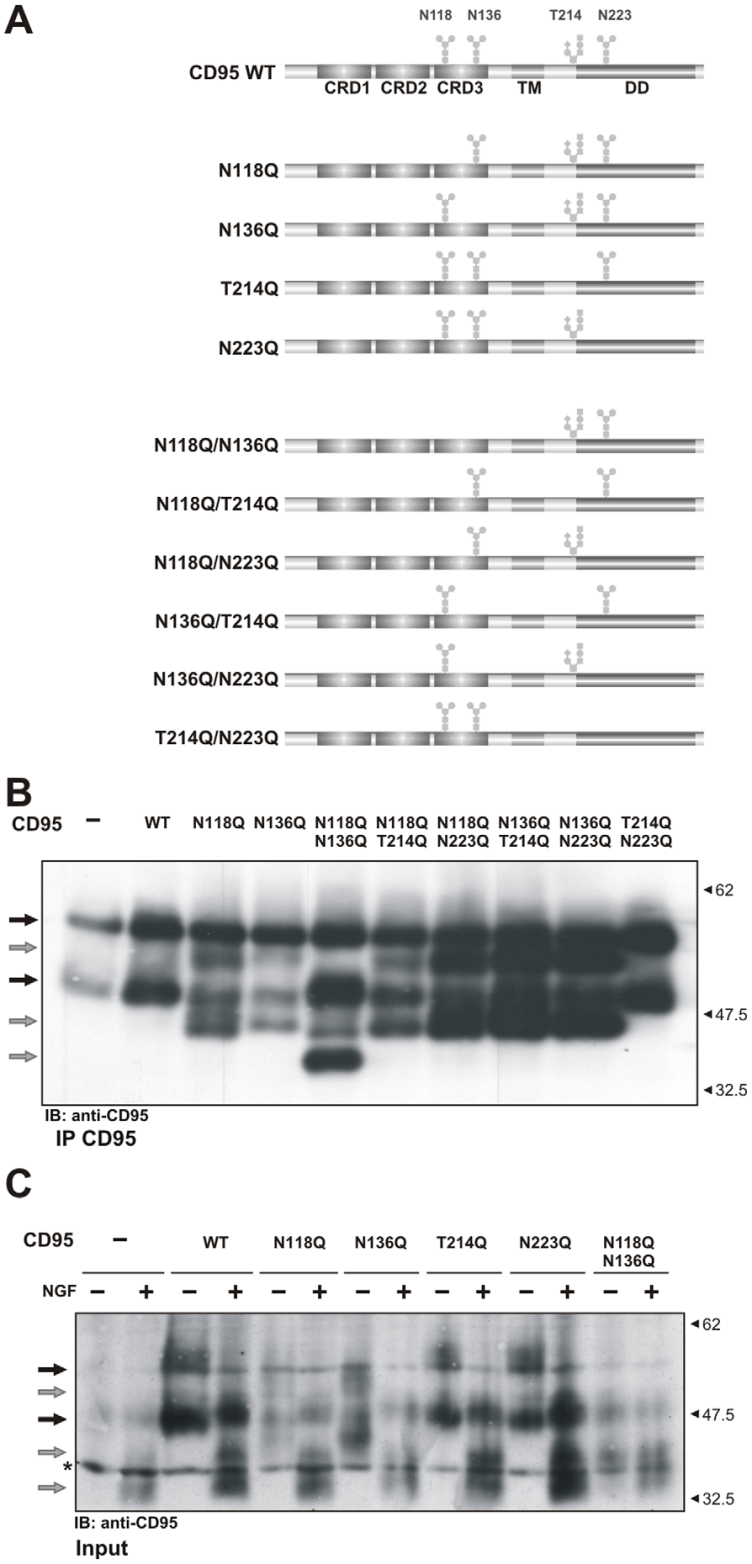
Analysis of CD95 glycosylation mutants. (A) Schematic overview of all generated glycomutants. (B) Expression of CD95 glycomutants in HeLa cells. CD95 pattern was analyzed by Western Blot using anti-CD95 polyclonal antibodies C20. WT CD95 bands are indicated by black arrows, while CD95 bands from glycomutants are indicated by grey arrows. (C) CD95 pattern in HeLa cells transfected with WT CD95 and CD95 glycomutants after N-glycosidase F treatment was analyzed by Western Blot using anti-CD95 polyclonal antibodies C20.

To confirm N-glycosylation of CD95 at the predicted glycosylation sites we generated CD95 single and double ‘glycomutants’ by site-directed mutagenesis (Figure 4A). Although glycan addition at the intracellular consensus site is highly unlikely, it was nevertheless included as a negative control for mutagenesis. Transient overexpression of these mutants in HeLa cells demonstrated that only glycomutants at positions N118 and N136 show characteristic CD95 band shifts to the lower molecular mass upon Western Blot analysis (Figure 4B). These changes in molecular mass, indicating a possible impaired glycosylation, were observed for single mutants N118Q and N136Q as well as for all double mutants containing N118Q and/or N136Q (Figure 4B). There were no CD95 bands shifts in case of the N223Q and T214Q mutants (Figure 4B). This demonstrates that there is no O- or N-glycosylation at the predicted sites in the intracellular domain of CD95 as expected. The treatment of all mutants with N-glycosidase F resulted in the characteristic shift of CD95 bands to the lower molecular mass (Figure 4C). Importantly, the introduction of the mutations at the glycosylation sites did not block transport of CD95 to the cell surface as monitored by flow cytometry cell surface staining (Figure S3A). Thus, site-directed mutagenesis indicated that two extracellular sites N118 and N136 are glycosylated.

### N-Glycosylation of CD95 does not play an essential role for caspase-8 activation at the DISC

**Figure 5:**
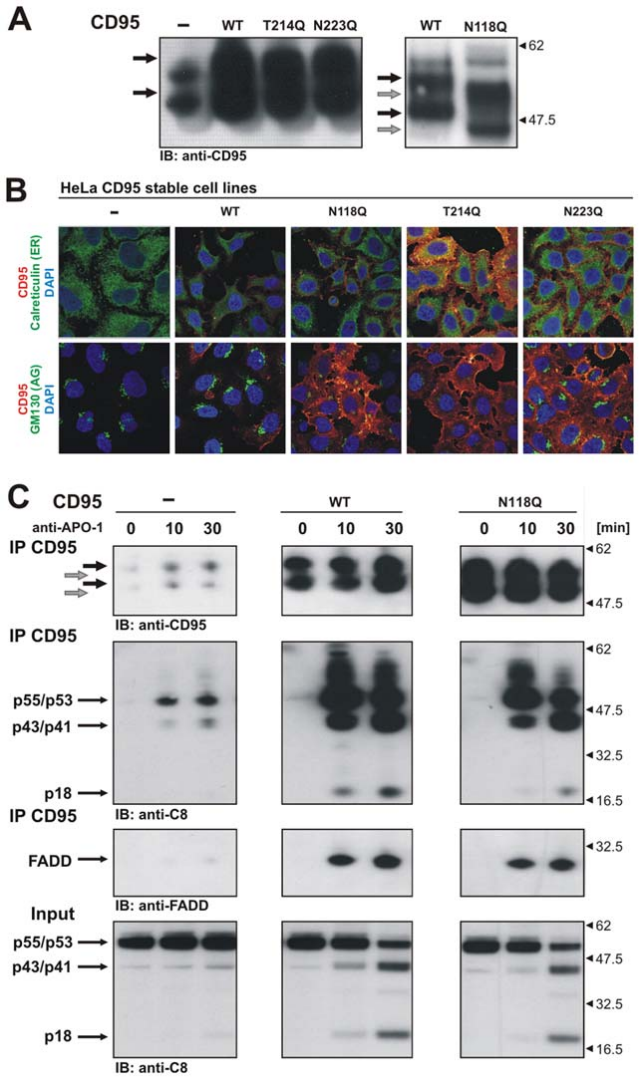
Analysis of HeLa-CD95 stable cell lines with CD95 glycosylation mutants. (A) CD95 protein pattern in cell lines stably expressing CD95 forms was analyzed by Western Blot analysis using anti-CD95 polyclonal antibodies C20. WT CD95 bands are indicated by black arrows, while CD95 bands from glycomutants are indicated by grey arrows. (B) HeLa cells stably transfected with WT CD95 and CD95 glycomutants were plated on glass slides, fixed, stained with anti-APO-1 antibodies for CD95, with anti-calreticulin for specific ER marker and with anti-GM130 for specific Golgi marker antibodies. Localization of CD95 was visualized by Alexa594-coupled secondary antibodies and localization of GM130 and calreticulin was visualized by Alexa488-coupled antibodies. Nuclei were stained with DAPI. (C) CD95 DISCs were analyzed after treatment with 500 ng/ml of anti-APO-1 antibodies for indicated time points. Western Blot analysis of the DISCs was performed with antibodies against CD95, procaspase-8 and FADD. CD95 bands in stably transfected HeLa-CD95 cells are indicated by black arrows, while for HeLa- CD95N118Q cells CD95 bands are indicated by grey arrows.

Having received evidence for a possible N-glycosylation at positions N118 and N136, we next addressed the influence of CD95 glycans on CD95 DISC formation. We therefore generated HeLa cell lines stably overexpressing WT CD95 (HeLa-CD95 cells) as well as different CD95 glycomutants (HeLa-CD95-N118Q, HeLa-CD95-N136Q, HeLa-CD95-T214Q, HeLa-CD95-N223Q cells). The level of endogenous CD95 was approximately 10 times lower than that of overexpressed WT CD95 as estimated by quantitative Western blot by Neumann and co-authors [26] (Figure 5A).

Plasma membrane targeting of CD95 is important for proper CD95/CD95L interactions and transduction of the apoptotic signal as shown earlier. In the generated stable HeLa-CD95 cell lines, CD95 appeared to be transported to the cell surface as monitored by flow cytometry cell surface staining (Figure S3B). Additional evidence for cell surface expression came from the confocal microscopy analysis of cellular compartmentalization of CD95 glycomutants. In these experiments CD95 glycomutants as well as WT CD95 could be observed on the plasma membrane (Figure 5B). These results confirmed that a CD95 containing mutation at individual glycosylation sites was translocated to the cellular membrane. In addition, we analyzed the stability of the glycomutants, *i. e.* N118Q, N136Q and N118Q/N136Q, using the inhibitor of translation, cyclohexamide (CHX) (Figure S3C). We did not observe any difference in the stability of CD95 glycomutants *vs.* WT CD95.

To analyze CD95 DISC formation and caspase-8 activation upon deglycosylation of CD95, HeLa-CD95 cells as well as HeLa-CD95 cells with glycomutants were stimulated with agonistic anti-APO-1-antibodies and the CD95 DISCs were immunoprecipitated and analyzed [27]. Analysis of the CD95 DISCs demonstrated that disruption of glycosylation by mutation (N118Q) did not influence FADD recruitment to the DISC (Figure 5C). The introduction of mutation at N136 also did not influence recruitment of FADD to the DISC (data not shown). However, we observed that procaspase-8a/b processing to its cleavage products p43/p41 and p18 at the DISC formed with the CD95-N118Q mutant occurred slightly slower than with WT CD95 (Figure 5C). After 10 min there was hardly any p18 detectable in HeLa-CD95-N118Q cells in comparison to HeLa-CD95 cells, even though the amount of immunoprecipitated CD95 was higher in HeLa-CD95-N118Q cells. This could not result from different affinities of WT CD95 and CD95-N118Q to anti-APO-1 antibodies because the affinity as demonstrated by ELISA analysis was similar for both WT and mutant (Figure S3D). Thus, we observed slower kinetics of procaspase-8 activation at the CD95 DISC in HeLa-CD95-N118Q cells. The experiments in HeLa cells stably transfected with CD95 glycomutants show that the disruption of glycosylation did not influence CD95 DISC formation, the recruitment of FADD to the DISC and only slightly slowed down procaspase-8 activation at the CD95 DISC.

To rule out that effects of deglycosylation of CD95 on caspase-8 activation have more impact upon the application of another stimuli of CD95, *e.g. via* CD95L, caspase-8 activation was compared upon stimulation with CD95L and anti-APO-1 antibodies. These experiments were carried out upon transient overexpression of WT CD95, N118Q, N136Q and N118Q/N136Q in HeLa cells (Figure S3E, F, G). Also CD95L also did not cause any significant increase or decrease of caspase-8 activation in glycomutant -transfected HeLa cells as compared to WT CD95-transfected HeLa cells.

Finally, as a number of reports [28] show the importance of stable, high molecular weight CD95 complexes for the efficient activation of caspase-8, we have analyzed whether these aggregates are perturbed after CD95 deglycosylation. We observed the formation of the high molecular weight complexes upon anti-APO-1 stimulation for HeLa cells with WT CD95, N118Q, N136Q and N118Q/N136Q (Figure S3H). Therefore, we have concluded that CD95 deglycosylation does not influence formation of these CD95 high molecular weight structures.

Nevertheless, the analysis of CD95 DISC in HeLa cells stably transfected with CD95 glycomutants has clear limitations due to the presence of endogenous CD95 albeit in lower amounts. Assessment of the effects induced by residual amounts of endogenous CD95 on DISC formation and caspase activation was not possible by this approach. Therefore, to analyze the CD95 DISC formation upon perturbation of CD95 N-glycosylation by other independent approaches cells were treated with VCN (recombinant *Vibrio cholerae* neuraminidase) and different inhibitors of N-glycosylation. This was followed by analysis of the DISC formation.

**Figure 6:**
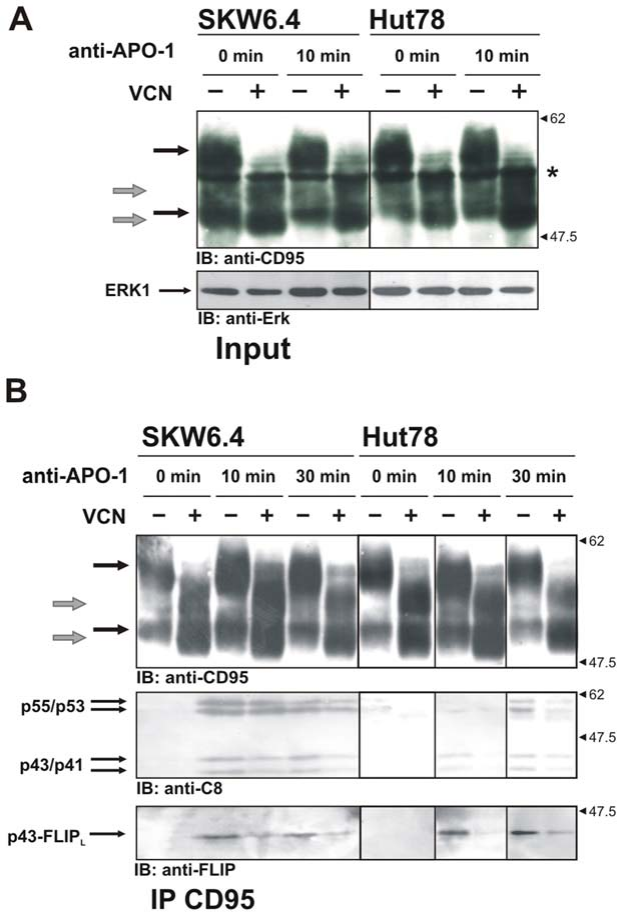
Analysis of CD95 DISC in cells treated with *Vibrio cholerae* Neuraminidase (VCN). (A) SKW6.4 and Hut78 cells were treated with VCN for 1 hour at 37°C. Total cellular lysates were analyzed using Western Blot with polyclonal antibodies C20. (B) SKW6.4 and Hut78 cells were treated with VCN for 1 hour at 37°C. CD95 DISCs were analyzed after treatment with 1 µg/ml of anti-APO-1 antibodies for indicated time points. Western Blot analysis of the DISCs was performed with antibodies against CD95, procaspase-8 and c-FLIP. CD95 bands in untreated cells are indicated by black arrows, while shifts of CD95 bands in VCN-treated cells are indicated by grey arrows.

VCN preferentially hydrolyzes linkages of sialic acid (Figure S2C). Treatment with VCN for one hour resulted in substantial desialylation of CD95 glycans, monitored by the shift to a lower molecular mass for both bands of CD95 (Figure 6A). To analyze CD95 DISC formation, CD95 DISCs were immunoprecipitated from untreated and VCN-treated SKW6.4 and Hut78 cells (Figure 6B). The CD95 DISC formed after VCN treatment had lower amounts of the procaspase-8a/b cleavage product p43/p41 and the cleavage product of c-FLIP_L_, p43-FLIP_L_ (Figure 6B).

To rule out that the reduced DISC formation after VCN treatment was not due to the decreased affinity of anti-APO-1 antibodies to desialylated CD95, we carried out an ELISA analysis and found that the binding of desialyated and sialylated CD95 to anti-APO-1 antibodies was similar (Figure S4A). In addition, no change of cell surface expression of CD95 was detected (Figure S4B). Thus, the diminished DISC formation was the consequence of VCN treatment.

We also tried to analyze CD95 DISC formation upon treatment with tunicamycin, an inhibitor of N-glycosylation that acts at the ER level blocking N-glycosylation (Figure S2D). However, the tunicamycin-induced N-deglycosylation was also accompanied by ER stress and inhibition of translation. This led to a decrease in c-FLIP levels, which are the main inhibitors of caspase-8 activation at the DISC. Therefore, the decrease in caspase-8 activation at the DISC could not be attributed to N-deglycosylation as the only cause (Figure S5A–C and data not shown).

**Figure 7:**
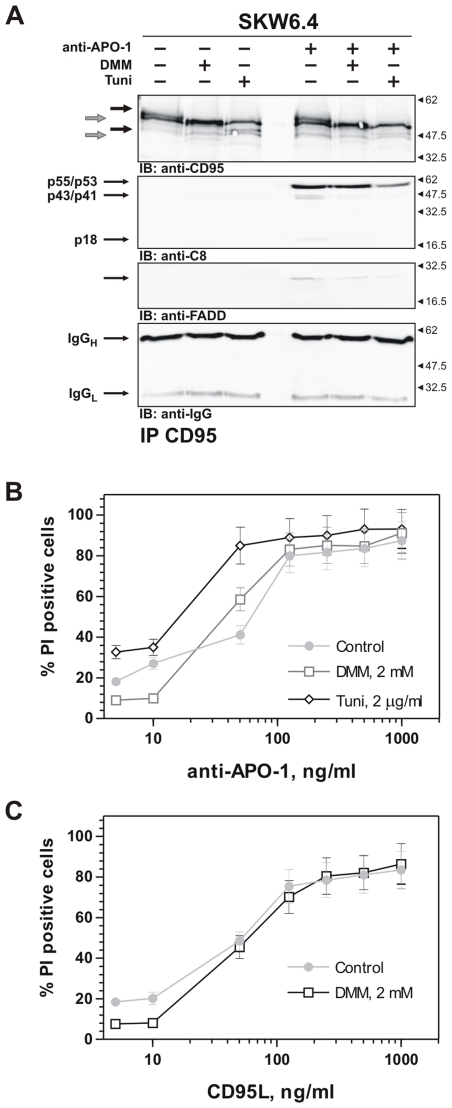
Analysis of the role of complex and hybrid glycans in CD95 signaling with DMM. (A) SKW6.4 were treated for 24 h with 2 mM DMM or 2 µg/ml of tunicamycin (Tuni) or left untreated. CD95 DISCs upon were analyzed after treatment with 500 ng/ml of anti-APO-1 antibodies for 15 min. Western Blot analysis of the DISCs was performed with antibodies against CD95, FADD and procaspase-8. IgG Western Blot was used as a loading control for immunoprecipitation. CD95 bands in non-treated cells are indicated by black arrows, while CD95 bands in DMM-treated cells are indicated by grey arrows. (B) and (C) Apoptotic cell death was measured with propidium iodide staining in SKW6.4 cells.

Finally, we also applied deoxymannojirimycin (DMM), a reagent that inhibits the ER mannosidases and Golgi mannosidase I, resulting in the accumulation of high-mannose oligosaccharide structures (Figure S2E). DMM treatment resulted in a shift to the lower molecular mass for both bands of CD95 in SKW6.4 cells pointing to the impairment of CD95 glycostructure (Figure 7A). Tunicamycin-treated cells were used in parallel to control CD95 band shifts. In DMM-treated cells CD95 was transported to the cell surface as shown by surface staining (Figure S6). Stimulation of CD95 with anti-APO-1 in DMM-treated cells resulted in decreased amounts of FADD at the DISC and diminished amounts of procaspase-8a/b processed at the CD95 DISC (Figure 7A).

Taken together, we showed that CD95 DISC is formed despite N-deglycosylation of CD95. Apparently N-deglycosylation of CD95 reduces the amount of active caspase-8 generated at the DISC.

### Deglycosylation of CD95 leads to decreased cell death under low strength CD95 stimulation conditions

After investigating procaspase-8 activation at the DISC we wanted to understand the contribution of CD95 N-glycosylation to the onset of apoptosis. The analysis of cell death upon CD95 deglycosylation has been complicated in the stable HeLa-CD95 cells as they contain endogenous CD95. The application of VCN and tunicamycin demonstrated that they alone were already toxic to the cells causing caspase activation and cell death (Figure S4C, D; Figure S5D, E and data not shown). As an alternative we also applied deoxymannojirimycin (DMM), a reagent with minimal toxicity to the cells [29]. Our experiments demonstrated that DMM treatment alone did not result in any cell death (Figure 7B). Interestingly, we observed that pretreatment with DMM caused a decrease in CD95-induced apoptosis upon stimulation with low amounts of CD95 agonists, *e.g*. anti-APO-1 and CD95L (Figure 7B and C). Upon stimulation with a high amount of CD95 agonists we did not observe any contribution of DMM pretreatment. This might be due to the fact that CD95 deglycosylation results in a small decrease of procaspase-8 processing at the DISC, which does not lead to significant effects on apoptosis provided the strength of stimuli and the rate of apoptosis are high. However, when the strength of stimulation is close to the threshold amount [5], [30] and the quantity of active caspase-8 generated at the DISC is low then alterations in caspase-8 amount can result in changes in the cell death level.

## Discussion

CD95-mediated apoptosis is one of the best-studied apoptotic signaling pathways. A number of studies have clarified the main CD95-mediated events: DISC formation, procaspase-8 activation and signaling in Type I and Type II cells [1], [31]. Despite this progress there are still many unknown details in the molecular mechanisms of CD95 signal transduction. In this study we aimed to understand the role of CD95 N-glycosylation in CD95 apoptotic signaling.

Using bioinformatic analysis we predicted the putative CD95 N-glycosylation sites at N118 and N136, which was confirmed by site-directed mutagenesis. *In silico* computer modeling showed that CD95 N-glycosylation might play a significant role in fine-tuning of the CD95 DISC complex. The modeling predicted that N136 may play an important role for CD95L/CD95 interactions and that N118 might be important for stabilization of the CD95 DISC network. Using biochemical analysis of the CD95 DISC on the HeLa-CD95 cells and cells treated with VCN and DMM we demonstrated that N-deglycosylation of CD95 slightly diminishes activation of procaspase-8 at the DISC. As we observed only small effects on procaspase-8 activation upon deglycosylation with DMM, which likely involves both N118 and N136, we did not follow the contribution of each residue, *e.g.* N118 or N136. From the modeling data we can suggest that both residues contribute to the decrease in procaspase-8 activation. Similar data were obtained upon deglycosylation of TRAIL-R by Wagner *et al.* [24]. Deglycosylation of TRAIL-R1 and TRAIL-R2 led to the diminished amounts of FADD and procaspase-8 at the TRAIL DISC, however, the effects were more drastic than upon deglycosylation of CD95 [24].

In contrast to the DISC analysis with N-deglycosylated CD95 it was more difficult to establish the optimal system for the analysis of the cell death. DMM treatment did not cause any toxicity to the cells, therefore, represented a reliable tool for the analysis of cell death mediated *via* deglycosylated CD95. We have found a reduction of CD95-induced apoptosis upon DMM pretreatment and low CD95 stimulation strength. This result corresponds quite well with the slightly diminished rate of procaspase-8 activation at deglycosylated CD95. These small kinetic effects could not dramatically change the outcome of cell death when the strength of CD95 stimulation was high. However, upon so-called threshold CD95 stimulation with low doses of CD95 antagonists [5], [30] the amount of CD95 DISCs formed is low and the amount of caspase-8 is just sufficient to trigger apoptosis [5], [30]. Therefore, a decrease in the amount of caspase-8 caused by deglycosylation of CD95 might result in apoptosis inhibition. Interestingly, deglycosylation of TRAIL-R1 and TRAIL-R2 results in more robust sensitisation towards apoptosis in contrast to CD95 deglycosylation. In the latter case the observed effects took place only under the narrow range of anti-CD95 concentrations.

Other methods of CD95 deglycosylation were found to be not appropriate for comparison of cell death mediated by N-deglycosylated CD95 and N-glycosylated CD95. HeLa-CD95 stable cell lines with different glycomutants contain endogenous CD95, which did not allow us to assess the contribution of only deglycosylated CD95 apoptosis induction. Tunicamycin induces a number of side effects, *e.g.* ER-stress and translation inhibition, that are toxic to the cells. The action of VCN resulted in the induction of CD95-independent cell death, complicating the analysis of CD95-induced apoptosis upon VCN action.

Interestingly, we have detected different forms of CD95 in various cell lines, which we showed not to be the result of differential N-glycosylation (Figure 2B, C). In some cells CD95 appears at Western Blots as two forms, 45 and 54 kDa. In other cells only one form, of appr. 50 kDa, is observed. Interestingly, most Type I cells, *e.g.* SKW6.4, Hut78 and BJAB, have two CD95 forms with a differential molecular mass of 9 kDa and Type II cells, *e.g.* Jurkat and CEM cells, mostly have one CD95 form. The nature of these forms is poorly understood. There might be two reasons for the presence of different receptor forms – alternative splicing and posttranslational modifications. We have shown that alternative splicing is not the reason for the presence of several CD95 protein forms in the cell lines under investigation (data not shown).

Several posttranslational modifications, *e.g.* N-glycosylation, proteolysis, C-mannosylation and palmitoylation, might lead to the significant shift in molecular mass of about 9 kDa and give rise to different forms of CD95. N-glycosylation was ruled out by our initial experiments. Treatment with N-glycosidase F showed that both CD95 forms shifted to a lower molecular mass. This is an important result as there are many reports which describe the different CD95 forms as a result of differential N-glycosylation [18], [26]. To analyze the possibility of C-mannosylation and palmitoylation we have performed bioinformatic analysis. This did not reveal any potential C-mannosylation sites with a probability score higher than 0.5 (data not shown) but demonstrated several potential palmitoylation sites. Recently, there has been a report that CD95 is a palmitoylated protein and that CD95 palmitoylation facilitates apoptosis induction [28]. However, palmitoylation of the protein does not result in a large difference in relative molecular mass using gel electrophoretic separation techniques and cells carrying mutations in the CD95 palmitoylated site did not have an altered mobility in SDS-PAGE [28]. This rules out the possibility that palmitoylation contributes to the generation of two forms of CD95. The possibility of proteolysis by the metalloproteinase matrilysin (MMP7) was also excluded as the reported cleavage of the extracellular domain of CD95 by MMP7 generates a small fragment of only one kDa [32]. The difference in molecular mass of the two CD95 forms is about 9 kDa, which excludes their generation by MMP7. However, we could not exclude proteolysis mediated by other proteinases as a possible reason for several forms of CD95 in some cell types. This question has to be addressed in future studies.

Recently, it has been reported that O-glycosylation of TRAILR1 (DR4) and TRAILR2 (DR5) plays a central role in regulation of sensitivity and resistance of cells towards TRAIL-induced apoptosis [24]. In our study we found that the only predicted O-glycosylation site in CD95 (T214) was not glycosylated. CD95 glycomutant T214Q showed the same CD95 protein pattern upon SDS-PAGE analysis as WT CD95. The glycosylation on T214Q was highly unlikely, as it is located in the intracellular domain. Another important result of our studies, as mentioned above, is that the influence of CD95 glycostructure on the apoptosis onset is less drastic than that for the TRAIL system. This might result from the different spatial organisation of the receptor complexes.

Taken together, using *in silico* modeling predictions together with biochemical approaches, we showed that glycostructure of CD95 can modulate procaspase-8 activation at the DISC. Furthermore, our findings provide evidence that the CD95 glycostructure contributes to the apoptotic signaling threshold defining cell death initiation. This may be additionally affected by different glycostructures. This regulation might be very important for cancer cells where subtle differences in the amount of caspase-8 regulate life or death of the cells.

## Materials and Methods

### Cell lines

The B lymphoblastoid cell lines SKW6.4 [33], BJAB [34] and the T cell lines Hut78 [35], CEM [36], Jurkat A3 [37], Jurkat 16 [38], Jurkat 27 [39] were maintained in RPMI 1640 (Life Technologies, Germany), 10 mM HEPES (Life Technologies, Germany), 50 µg/ml Gentamycin (Life Technologies, Germany) and 10% fetal calf serum (Life Technologies, Germany) in 5% CO_2_. HeLa [40] and HEK293T [41] cell lines were maintained in DMEM (Life Technologies, Germany), 0.5% Penicillin-Streptomycin (Life Technologies, Germany) and 10% fetal calf serum (Life Technologies, Germany) in 5% CO_2_.

### Antibodies and reagents

Anti-CD95 polyclonal antibodies C20 were purchased from Santa Cruz Biotechnology (Heidelberg, Germany). The anti-FADD mAb 1C4 (mouse IgG1) recognizes the C-terminus of FADD [42]. The anti-FLIP mAb NF6 (mouse IgG1) recognizes the N-terminus of FLIP [43]. The anti-caspase-8 mAb C15 (mouse IgG2b) recognizes the p18 subunit of caspase-8 [11]. Anti-APO-1 is an agonistic monoclonal antibody recognizing an epitope on the extracellular part of CD95 (APO-1/Fas) [4]. Anti-tubulin antibodies were purchased from Sigma. Anti-ERK antibodies were from BD Transduction Laboratories. Anti-JNK antibodies were purchased from Santa-Cruz Biotechnology. Horseradish peroxidase-conjugated goat anti-mouse IgG1, -2a and -2b were from Southern Biotechnology Associates (United Kingdom). The coding sequence of LZ-CD95L [44] was cloned into a pIRESpuro3 plasmid (Clontech, France). Recombinant LZ-CD95L was produced using 293T cells stably transfected with this vector. Tunicamycin and DMM were purchased from Calbiochem (Darmstadt, Germany); VCN was from Sigma-Aldrich (Germany). N-glycosidase F was from Roche (Mannheim, Germany). CHX was from Sigma (Germany). pKEX plasmid was published in [45]. All other chemicals used were of analytical grade and purchased from Merck (Germany) or Sigma (Germany).

### Flow Cytometry analysis

The percentage of viable cells was determined by FSC/SSC and propidium iodide staining using a FACScallibur Cytometer (BD). A minimum of 10000 cells per sample was analyzed.

#### Surface staining

To analyze the surface expression of CD95, 5×10^5^ cells were resuspended in 100 µl of FACS buffer (10% FCS in PBS) and incubated with 10 µg/ml of anti-APO-1 antibodies or with FII23 antibodies as isotype control for 15 min on ice. The cells were washed with FACS buffer, centrifuged and resuspended in 100 µl of FACS buffer containing PE-conjugated anti-mouse IgG antibody and incubated on ice for 15 min. The cells were washed with FACS buffer and resuspended in 300 µl of FACS buffer containing 1 µg/ml PI. The staining was analyzed by flow cytometry. The population was gated on living cells and the staining of isotype control was compared to the surface staining with anti-APO-1 antibody.

### Preparation of total cellular lysates

1×10^8^ or 1×10^6^ cells were washed twice in 1× PBS and subsequently lysed in the lysis buffer (20 mM Tris/HCl, pH 7.5, 150 mM NaCl, 2 mM EDTA, 1 mM phenylmethylsulfonyl fluoride (Sigma, Germany), protease inhibitor cocktail (Roche, Switzerland), 1% Triton X-100 (Serva, Germany) and 10% glycerol) (stimulation condition) or lysed without treatment (unstimulated). The total cellular lysates were subsequently analyzed by Western Blot.

### DISC analysis by immunoprecipitation and Western Blot

1×10^8^ cells were treated with 1 µg/ml of anti-APO-1 antibodies at 37°C for indicated periods of time, washed twice in 1× PBS and subsequently lysed in the lysis buffer (stimulation condition) or lysed without treatment (unstimulated). The CD95 DISC was immunoprecipitated overnight with 2 µg of anti-APO-1 and protein A sepharose beads. Protein A sepharose beads were washed five times with 20 volumes of lysis buffer. The immunoprecipitates were analyzed on 12% PAAG. Subsequently, the gels were transferred to Hybond nitrocellulose membrane (Amersham Pharmacia Biotech., Germany), blocked with 5% nonfat dry milk in PBS/Tween (PBS plus 0.05% Tween 20) for 1 h, washed with PBS/Tween, and incubated with the primary antibodies in PBS/Tween at 4°C overnight. Blots were developed with a chemoluminescence method following the manufacturer's protocol (Perkin Elmer Life Sciences, Germany).

### Glycosidase treatment

For desialylation, 1×10^7^ cells were washed twice in 1× PBS and then treated with 100 mU VCN in RPMI, pH 6.8 for 1 hour at 37°C. For N-glycosidase F treatment 10^7^ cells were lysed in buffer A and, subsequently the total cellular lysates were treated with N-glycosidase F following the protocol by the manufacturer.

### Inhibition of glycosylation

To completely inhibit N-linked glycosylation cells were cultured for 20 hours in medium containing 2 µg/ml of tunicamycin. For inhibition of mannosidase-1 cells were cultured for 48 hours in medium containing 2 mM DMM.

### Inhibition of translation

For inhibition of protein translation cells were cultured for 0, 3 and 9 hours in medium containing 10 µg/ml of CHX.

### Enzyme-linked immunosorbent assay (ELISA)

Flexible 96-well plates from Becton Dickinson (USA) were coated with 100 µl of anti-CD95 (Abcam) antibody in 0.05 M carbonate-bicarbonate buffer pH 9.6 (CBB) at 4°C overnight. Then the plates were washed 3 times with distilled water, blocked with 200 µl of PBS containing 3% bovine serum albumin (BSA) at RT for 2 hours, which was followed by washing 3 times with PBS containing 0.5% Tween 20 (PBS-T). 100 µl of total cell lysates were added to each well, which was followed by incubation at RT for 1 h. After incubation, the plates were washed 3 times with PBS-T. This was followed by addition of 100 µl of peroxidase-conjugated goat anti-mouse IgG antibody (Jackson ImmunoResearch Laboratories) diluted at 1/5000 in PBS-1% BSA. The plates were incubated for 1 h at 4°C. After incubation, the plates were washed 3 times with PBS-T and then the reaction was revealed with 100 µl of OPD 0.4 mg/ml (Sigma) in 0.05 M phosphate-citrate buffer, pH 5.0 solution for 10–20 min at room temperature. After stopping the reaction with 100 µl of 3 N H_2_SO_4_, the plates were read with an ELISA reader (Wallac, Gaithersburg, USA) at 490 nm.

### Homology modeling and structures visualization

Modeling was carried out as previously described in [46], [47] using the MODELLER package [48]. Human CD95 ECD was modeled using crystal structures of tumor necrosis factor receptor (PDB ID 1ncf [49] and 1tnr.R [50]) as the templates. The CD95 transmembrane region was modeled as an α-helix bases on the prediction of the TMHMM Server v. 2.0 (www.cbs.dtu.dk/services/TMHMM/). To model free full-length CD95 receptor as envisaged on a plasma membrane the CD95 ECD and TM domains were modeled as described above and the model of CD95 DD domain was based on the NMR structure of CD95 DD in solution (PDB ID 1ddf, [51]) added. Human CD95L ECD was modeled using the crystal structure of tumor necrosis factor beta (PDB ID 1tnr.A [50]) as the template. The model of the complete CD95 DISC core structure was assembled on the crystal structure of Apo2L/TRAIL in a complex with death receptor 5 (PDB ID 1dog [52]): CD95 ECD and TM domains were modeled as described above and the model of the CD95 DD domain was based on the CD95 DD crystal structure from the CD95/FADD DDs complex (PDB ID 3ezq [16]). As their precise composition and structure is unknown, only core structures of N-glycans were added using GlyProt [53]. GROMACS [54] molecular dynamics and the quality analysis (ANOLEA [55], VERIFY_3D and ERRAT [http://nihserver.mbi.ucla.edu/]) and visualization/analysis (SwissPBD Viewer [56] and PyMol [www.pymol.org]) tools were employed as described in details in [46], [47] use instead [57].

## Supporting Information

Figure S1 **Alignment of CD95 sequences from different organisms.** (A) ClustalX2 sequence alignment of CD95 ECD across different species. Sequence abbreviations: Homo sapiens (HUMAN); Macaca fascicularis (MACFA); Macaca nemestrina (MACNE); Cercocebus torquatus (CERTO); Macaca mulatta (MACMU); Macaca arctoides (MACAR); Macaca assamensis (MACAS); Aotus trivirgatus (AOTTR); Callithrix jacchus (CALJA); Oryctolagus cuniculus (RABIT); Sus scrofa (PIG); Ovis aries (SHEEP); Felis catus (FELCA); Bos taurus (BOVIN); Mus musculus (MOUSE) and Rattus norvegicus (RAT). Sequences from NCBI Protein databank. Color lines on the top of the alignment indicate cysteine residues forming disulphide bonds in human CD95. N-X-S/T sequons are indicated by red boxes. (B) Probability of glycosylation (glycosylation potential) of sequons at positions 118 and 136 in human CD95 and their analogues in CD95 from other species was calculated by NetNGlyc 1.0 server.
(TIF)

Figure S2 **Schematic mechanisms of enzymatic and inhibitory deglycosylation.** (A) Core structure of N-glycan added to the models is a minimal possible composition of N-glycans. (B) The mechanism of action of N-glycosidase F. Variable structures of glycan side chains are presented in grey. (C) The mechanism of action of tunicamycin. Variable structures of glycan side chains are presented in grey. (D) The mechanism of action of VCN. Variable structures of glycan side chains are presented in grey. (E) The mechanism of action of DMM. Variable structures of glycan side chains are presented in grey.
(TIF)

Figure S3 **Analysis of CD95 glycosylation mutants.** (A) Cell surface staining of WT CD95 and CD95 glycomutants for transiently transfected HeLa cells was performed with anti-APO-1 IgG3 antibodies (red line). As isotype control FII23C IgG3 antibodies were used (Black and violet lines). To control efficiency of CD95 surface expression HeLa-CD95 stable cell line was used (green line). (B) Cell surface staining of WT CD95 and CD95 glycomutants in stable cell lines was performed with anti-APO-1 IgG3 antibodies. As isotype control FII23C IgG3 antibodies were used. (C) Degradation of WT CD95 and CD95 glycomutants in transiently transfected HEK293T cells was performed upon treatment with CHX. Total cellular lysates were analyzed after treatment with CHX using Western Blot with polyclonal antibodies C20, monoclonal NF6 antibodies against FLIP and anti-tubulin antibodies.(D) Binding of CD95 WT and glycomutants to anti-APO-1 antibodies. Cellular lysates from the HeLa cells stably transfected with CD95 WT and glycomutants were used for anti-APO-1-specific ELISA analysis. Anti-APO-1 was used in concentrations. 0.1, 0.2, 0.4, 0.8 and 1 µg/ml. (E) and (F). Comparison of caspase-8 activation was performed upon treatment with CD95L or anti-APO-1. To control activation of caspase-8 transiently transfected with WT CD95 and CD95 glycomutants HeLa cells were stimulated with CD95L or anti-APO-1 for indicated time points. Total cellular lysates were analyzed using Western Blot with C15 monoclonal antibodies for caspase-8 and anti-tubulin antibodies. (G). Control of CD95 expression for (E) and (F) was done after immunoprecipitation with anti-APO-1 by Western Blot with C20 polyclonal antibodies. (H). The ability to form CD95n oligomeric structures was compared between WT CD95 and CD95 glycomutants. Analysis was done by Western Blot with polyclonal C20 antibodies and anti-ERK antibodies. In the (C), (G) and (H) WT CD95 bands are indicated by black arrows, while CD95 bands from glycomutants are indicated by grey arrows. CD95n oligomeric structures are indicated by red arrow.
(TIF)

Figure S4 **Analysis of deglycosylation of CD95 with VCN. (**A) ELISA analysis for the binding of anti-APO-1 antibodies to CD95 from the lysates of untreated and VCN-treated SKW6.4 cells. (B) Cell surface staining of untreated and VCN-treated SKW6.4 cells was performed with anti-APO-1 IgG3 antibodies. As isotype control FII23C IgG3 antibodies were used. (C) Caspase-3 processing and Bid cleavage were analyzed in untreated and VCN-treated SKW6.4 cells using Western Blot. (D) SKW6.4 cells were treated as it was described in A and cell death was measured with propidium iodide staining.
(TIF)

Figure S5 **Analysis of CD95 N-glycosylation with tunicamycin.** (A) SKW6.4 and Hut78 cells were treated for 24 h with 2 µg/ml of tunicamycin (Tuni) or left untreated. CD95 DISCs were analyzed after stimulation with 500 ng/ml of anti-APO-1 antibodies for indicated time points. Western Blot analysis of the DISCs was performed with antibodies against CD95, procaspase-8 and c-FLIP. CD95 bands in non-treated cells are indicated by black arrows, while shifts of CD95 bands in tunicamycin-treated cells are indicated by grey arrows. (B) Cell surface staining of CD95 was performed with anti-APO-1 IgG3 antibodies. As isotype control FII23C IgG3 antibodies were used. (C) C-FLIP expression was analyzed by Western Blot analysis using monoclonal NF6 antibodies. (D) SKW6.4 and Hut78 cells were treated for 24 h with 2 µg/ml of tunicamycin (Tuni) or left untreated. Total cellular lysates were analyzed after treatment with 1 µg/ml of anti-APO-1 antibodies using Western Blot with polyclonal antibodies C20 and monoclonal antibodies C15 against procaspase-8. Anti-JNK1 Western Blot was used as a loading control. (E) SKW6.4 cells were treated as was described in A and apoptotic cell death was measured with propidium iodide staining.
(TIF)

Figure S6 **The analysis of deglycosylation of CD95 with DMM.** (A) Cell surface staining of CD95 was performed with anti-APO-1 IgG3 antibodies. As isotype control FII23C IgG3 antibodies were used.
(TIF)
