# Supplementary figures and images for: Modulation of the CD95-Induced Apoptosis: The Role of CD95 N-Glycosylation
Source: PLoS One. 2011 May 18;6(5):e19927. doi: 10.1371/journal.pone.0019927 (PMC3097226; doi:10.1371/journal.pone.0019927)

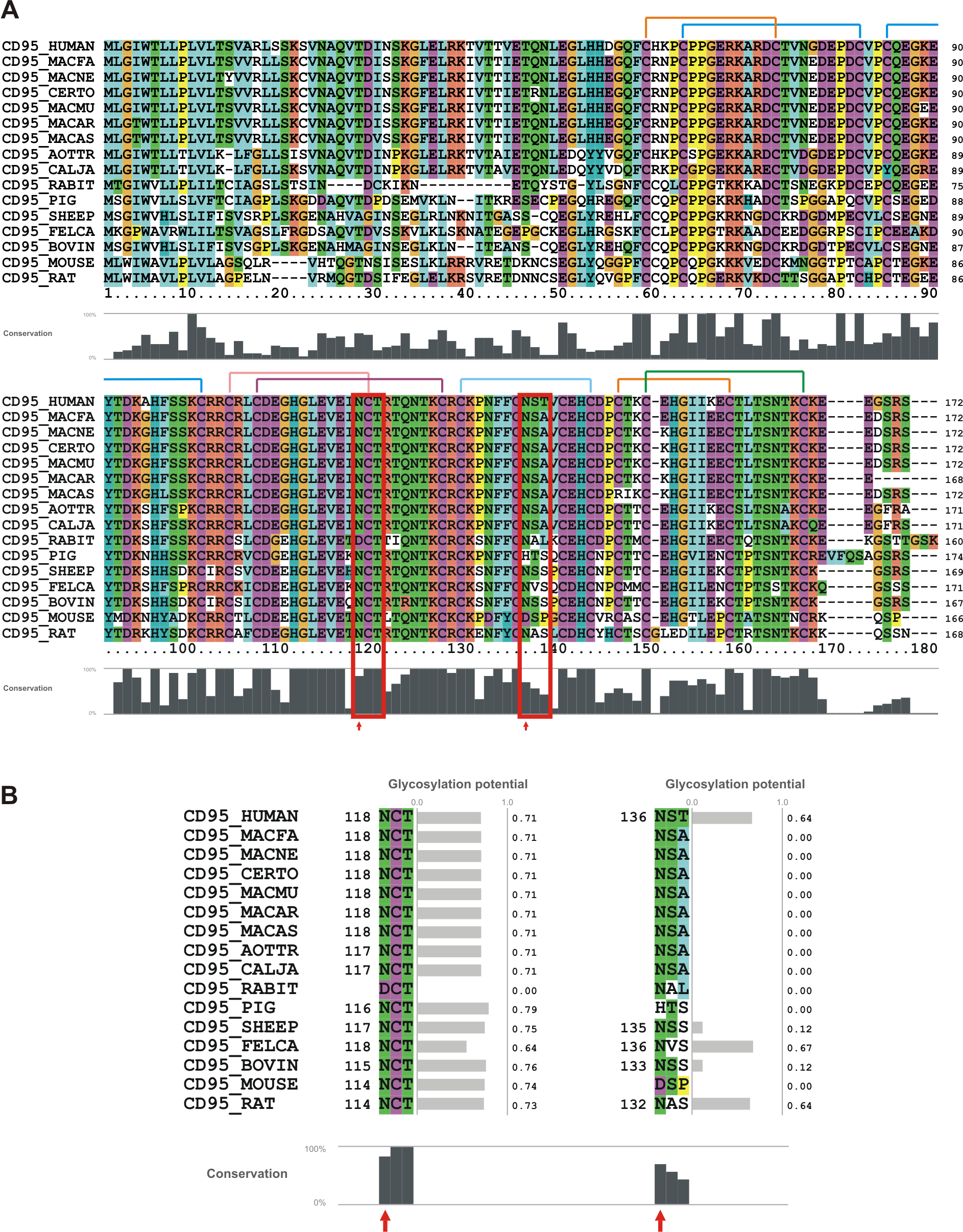

Supplement: Figure S1 — Alignment of CD95 sequences from different organisms. (A) ClustalX2 sequence alignment of CD95 ECD across different species. Sequence abbreviations: Homo sapiens (HUMAN); Macaca fascicularis (MACFA); Macaca nemestrina (MACNE); Cercocebus torquatus (CERTO); Macaca mulatta (MACMU); Macaca arctoides (MACAR); Macaca assamensis (MACAS); Aotus trivirgatus (AOTTR); Callithrix jacchus (CALJA); Oryctolagus cuniculus (RABIT); Sus scrofa (PIG); Ovis aries (SHEEP); Felis catus (FELCA); Bos taurus (BOVIN); Mus musculus (MOUSE) and Rattus norvegicus (RAT). Sequences from NCBI Protein databank. Color lines on the top of the alignment indicate cysteine residues forming disulphide bonds in human CD95. N-X-S/T sequons are indicated by red boxes. (B) Probability of glycosylation (glycosylation potential) of sequons at positions 118 and 136 in human CD95 and their analogues in CD95 from other species was calculated by NetNGlyc 1.0 server. (TIF) [file pone.0019927.s001.tif]

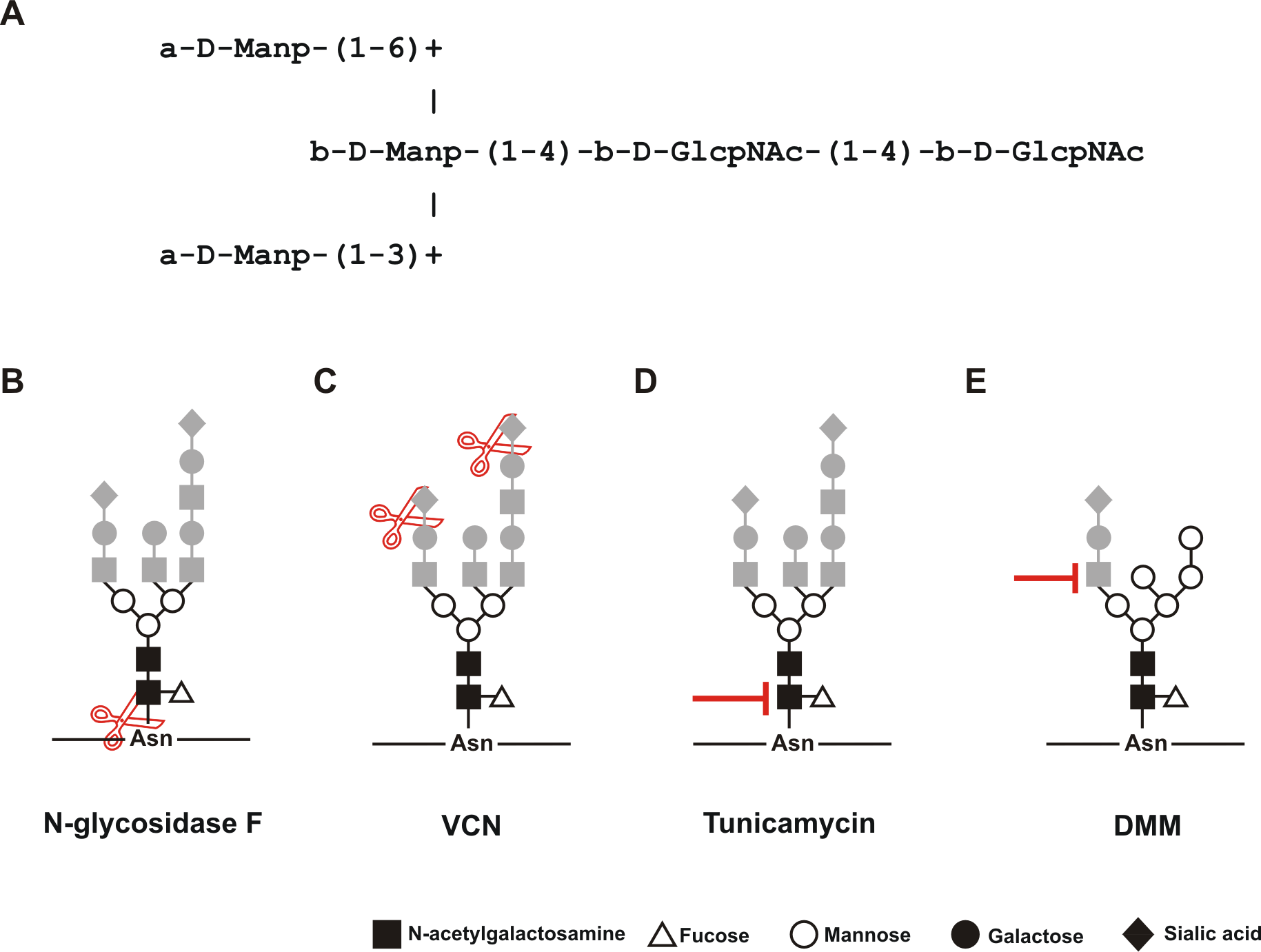

Supplement: Figure S2 — Schematic mechanisms of enzymatic and inhibitory deglycosylation. (A) Core structure of N-glycan added to the models is a minimal possible composition of N-glycans. (B) The mechanism of action of N-glycosidase F. Variable structures of glycan side chains are presented in grey. (C) The mechanism of action of tunicamycin. Variable structures of glycan side chains are presented in grey. (D) The mechanism of action of VCN. Variable structures of glycan side chains are presented in grey. (E) The mechanism of action of DMM. Variable structures of glycan side chains are presented in grey. (TIF) [file pone.0019927.s002.tif]

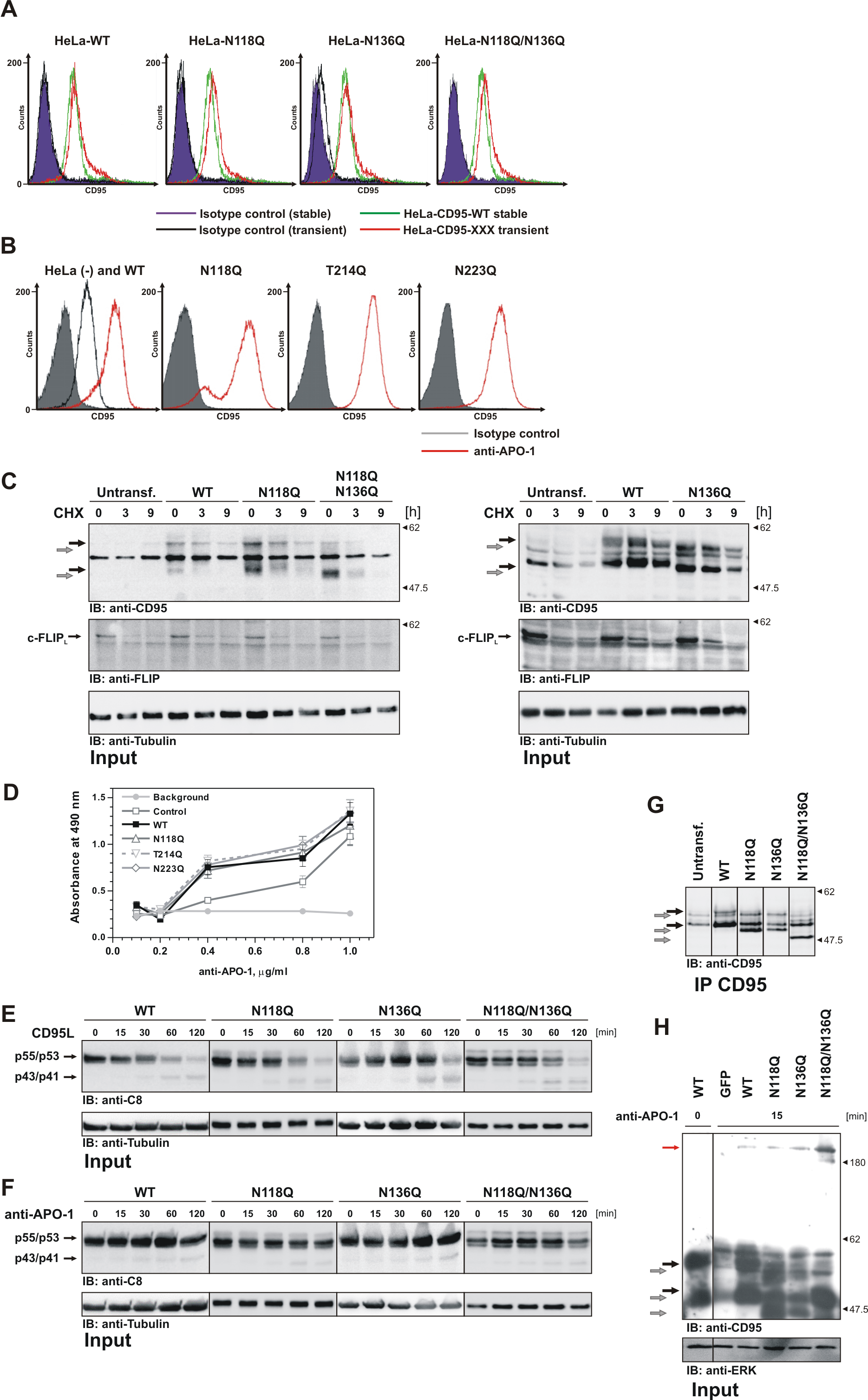

Supplement: Figure S3 — Analysis of CD95 glycosylation mutants. (A) Cell surface staining of WT CD95 and CD95 glycomutants for transiently transfected HeLa cells was performed with anti-APO-1 IgG3 antibodies (red line). As isotype control FII23C IgG3 antibodies were used (Black and violet lines). To control efficiency of CD95 surface expression HeLa-CD95 stable cell line was used (green line). (B) Cell surface staining of WT CD95 and CD95 glycomutants in stable cell lines was performed with anti-APO-1 IgG3 antibodies. As isotype control FII23C IgG3 antibodies were used. (C) Degradation of WT CD95 and CD95 glycomutants in transiently transfected HEK293T cells was performed upon treatment with CHX. Total cellular lysates were analyzed after treatment with CHX using Western Blot with polyclonal antibodies C20, monoclonal NF6 antibodies against FLIP and anti-tubulin antibodies.(D) Binding of CD95 WT and glycomutants to anti-APO-1 antibodies. Cellular lysates from the HeLa cells stably transfected with CD95 WT and glycomutants were used for anti-APO-1-specific ELISA analysis. Anti-APO-1 was used in concentrations. 0.1, 0.2, 0.4, 0.8 and 1 µg/ml. (E) and (F). Comparison of caspase-8 activation was performed upon treatment with CD95L or anti-APO-1. To control activation of caspase-8 transiently transfected with WT CD95 and CD95 glycomutants HeLa cells were stimulated with CD95L or anti-APO-1 for indicated time points. Total cellular lysates were analyzed using Western Blot with C15 monoclonal antibodies for caspase-8 and anti-tubulin antibodies. (G). Control of CD95 expression for (E) and (F) was done after immunoprecipitation with anti-APO-1 by Western Blot with C20 polyclonal antibodies. (H). The ability to form CD95n oligomeric structures was compared between WT CD95 and CD95 glycomutants. Analysis was done by Western Blot with polyclonal C20 antibodies and anti-ERK antibodies. In the (C), (G) and (H) WT CD95 bands are indicated by black arrows, while CD95 bands from glycomutants are indicated [file pone.0019927.s003.tif]

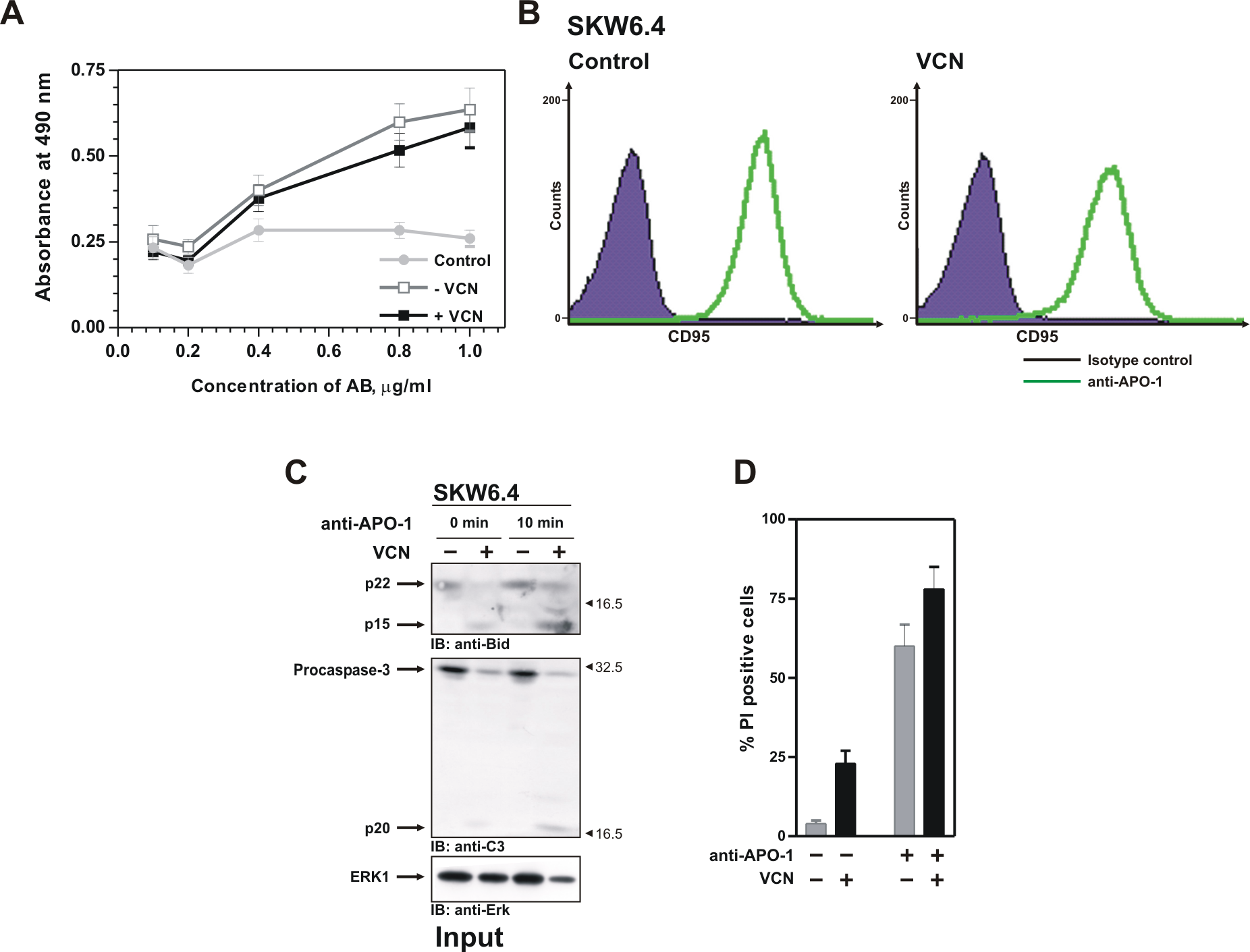

Supplement: Figure S4 — Analysis of deglycosylation of CD95 with VCN. (A) ELISA analysis for the binding of anti-APO-1 antibodies to CD95 from the lysates of untreated and VCN-treated SKW6.4 cells. (B) Cell surface staining of untreated and VCN-treated SKW6.4 cells was performed with anti-APO-1 IgG3 antibodies. As isotype control FII23C IgG3 antibodies were used. (C) Caspase-3 processing and Bid cleavage were analyzed in untreated and VCN-treated SKW6.4 cells using Western Blot. (D) SKW6.4 cells were treated as it was described in A and cell death was measured with propidium iodide staining. (TIF) [file pone.0019927.s004.tif]

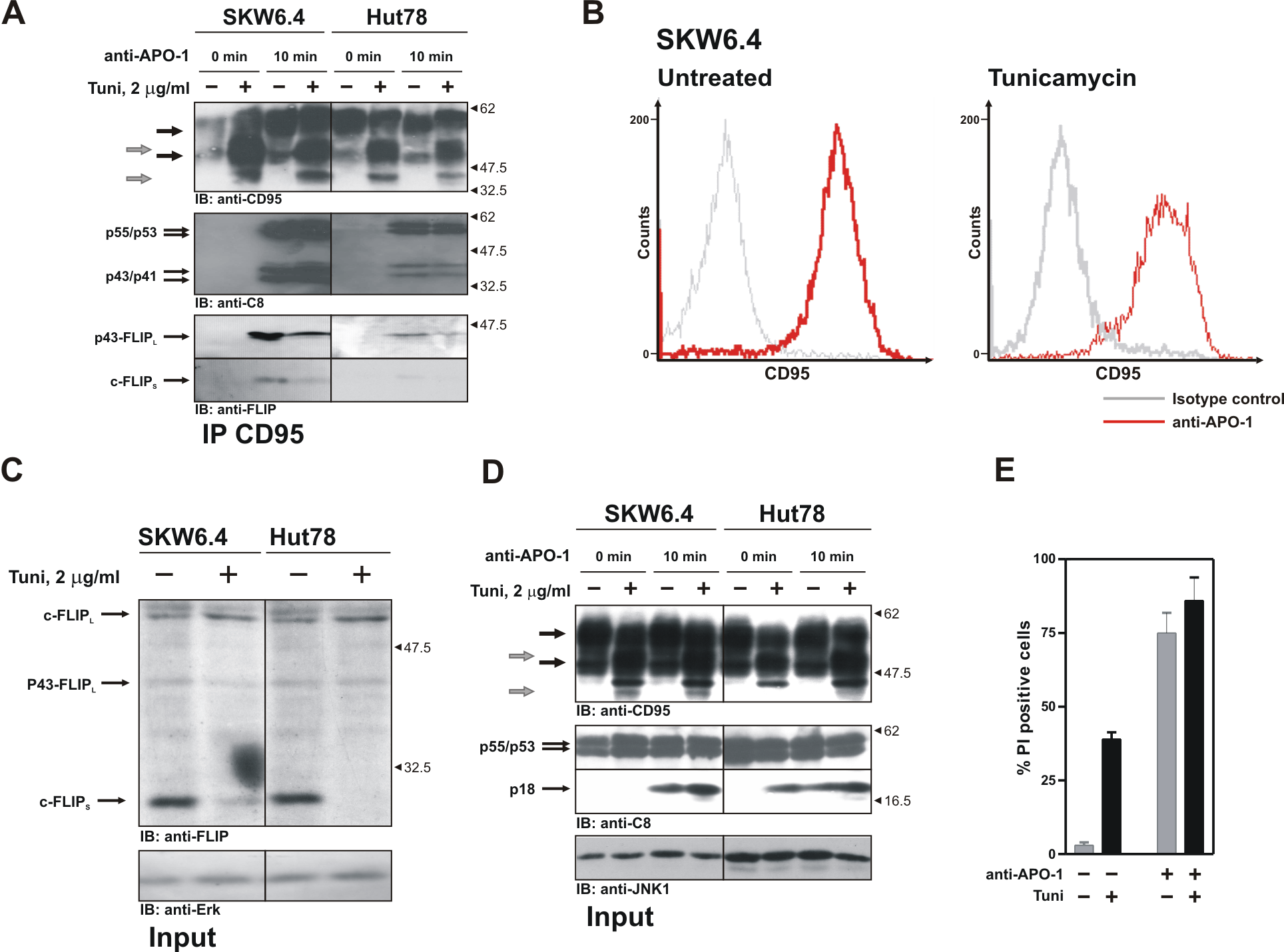

Supplement: Figure S5 — Analysis of CD95 N-glycosylation with tunicamycin. (A) SKW6.4 and Hut78 cells were treated for 24 h with 2 µg/ml of tunicamycin (Tuni) or left untreated. CD95 DISCs were analyzed after stimulation with 500 ng/ml of anti-APO-1 antibodies for indicated time points. Western Blot analysis of the DISCs was performed with antibodies against CD95, procaspase-8 and c-FLIP. CD95 bands in non-treated cells are indicated by black arrows, while shifts of CD95 bands in tunicamycin-treated cells are indicated by grey arrows. (B) Cell surface staining of CD95 was performed with anti-APO-1 IgG3 antibodies. As isotype control FII23C IgG3 antibodies were used. (C) C-FLIP expression was analyzed by Western Blot analysis using monoclonal NF6 antibodies. (D) SKW6.4 and Hut78 cells were treated for 24 h with 2 µg/ml of tunicamycin (Tuni) or left untreated. Total cellular lysates were analyzed after treatment with 1 µg/ml of anti-APO-1 antibodies using Western Blot with polyclonal antibodies C20 and monoclonal antibodies C15 against procaspase-8. Anti-JNK1 Western Blot was used as a loading control. (E) SKW6.4 cells were treated as was described in A and apoptotic cell death was measured with propidium iodide staining. (TIF) [file pone.0019927.s005.tif]

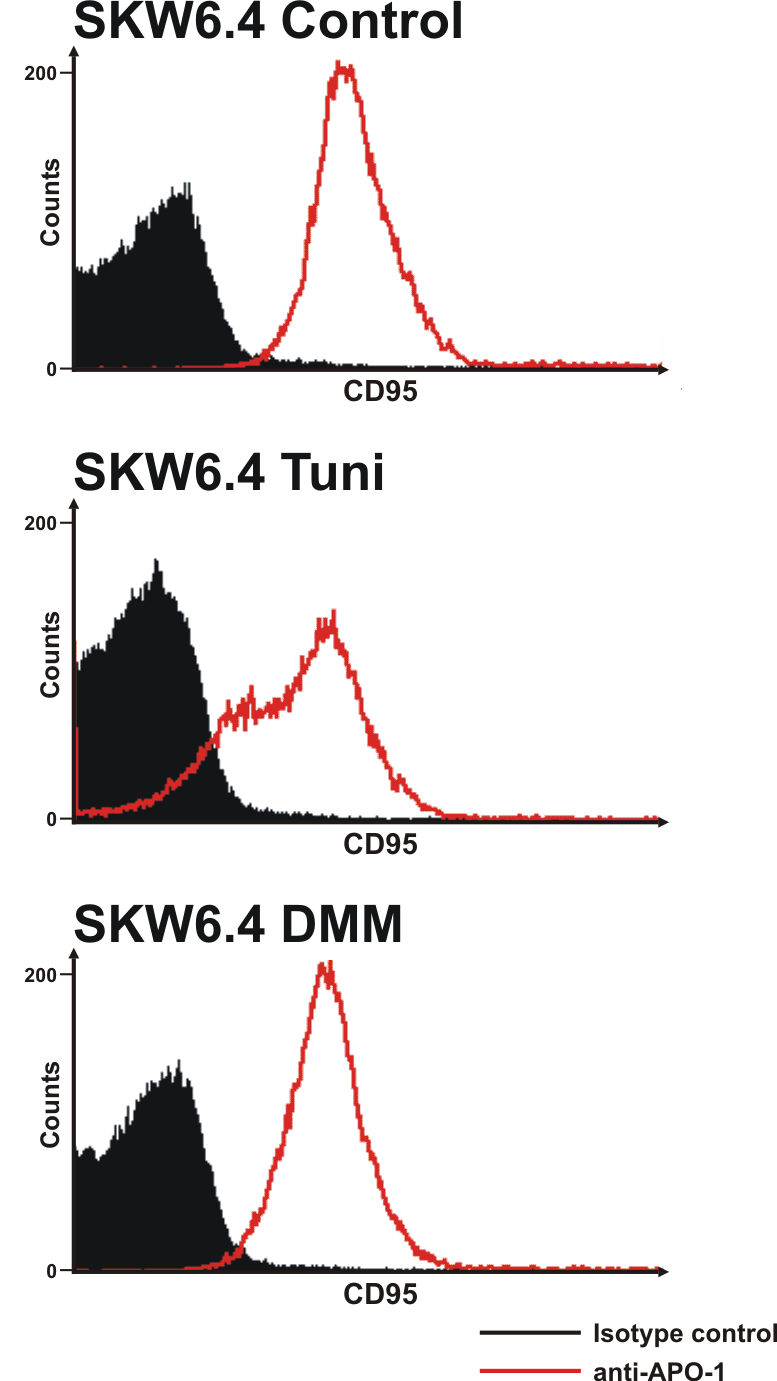

Supplement: Figure S6 — The analysis of deglycosylation of CD95 with DMM. (A) Cell surface staining of CD95 was performed with anti-APO-1 IgG3 antibodies. As isotype control FII23C IgG3 antibodies were used. (TIF) [file pone.0019927.s006.tif]
